# Prevalence of Poor Sleep Quality and Its Association with Dysmenorrhea Among Female Undergraduate Students at a Health Sciences University in the UAE

**DOI:** 10.3390/healthcare14040474

**Published:** 2026-02-13

**Authors:** Shadha Nasser Bahutair, Rajani Dube, Anishika Gnanadhas, Fathima Masharifa, Lianta Linus, Mohamed Ahmed Mohamed, Mohamedanas Mohamedfaruk Patni, Taliaa Mohsen Qasem Al-Yafeai, Shaimaa Hashem Elsalous

**Affiliations:** 1 RAK College of Medical Sciences, Ras Al Khaimah Medical and Health Sciences University, Ras Al Khaimah P.O. Box 11172, United Arab Emirates; rajnidube@yahoo.com (R.D.); anishika.20901080@rakmhsu.ac.ae (A.G.); fathima.20901007@rakmhsu.ac.ae (F.M.); lianta.21901074@rakmhsu.ac.ae (L.L.); mohamed.21901029@rakmhsu.ac.ae (M.A.M.); mohamedanas@rakmhsu.ac.ae (M.M.P.); 2 RAK College of Nursing, Ras Al Khaimah Medical and Health Sciences University, Ras Al Khaimah P.O. Box 11172, United Arab Emirates; taliaa@rakmhsu.ac.ae (T.M.Q.A.-Y.); shaimaa.hashem@rakmhsu.ac.ae (S.H.E.)

**Keywords:** menstrual pain, sleep disturbance, functional impairment, university students, women’s health, United Arab Emirates, Middle East, Arab population

## Abstract

**Background:** Poor sleep quality is common among university students and may contribute to adverse reproductive health outcomes, including dysmenorrhea. However, limited evidence exists on whether chronic sleep disturbance independently predicts dysmenorrhea severity or menstrual-related functional impairment after accounting for key confounders. **Objectives:** We aimed to determine the prevalence of poor sleep quality among female university students and to examine its association with (1) severe dysmenorrhea and (2) menstrual-related functional impairment. **Methods:** A cross-sectional study was conducted among female undergraduate students at Ras Al Khaimah Medical and Health Sciences University (United Arab Emirates). Sleep quality was measured using the Pittsburgh Sleep Quality Index (PSQI), and perceived stress was assessed using the Perceived Stress Scale (PSS-10). Dysmenorrhea severity was assessed using a 0–10 visual analog scale; functional impairment was defined as moderate/severe disruption in ≥1 life domain. Multivariable logistic regression models estimated adjusted odds ratios (aORs) for the association between sleep quality and menstrual outcomes after controlling for age, BMI, socioeconomic status, and stress. A component-level analysis examined independent effects of PSQI dimensions. **Results:** Of the 254 participants, 68.9% reported poor sleep quality and 48.8% reported severe dysmenorrhea. In adjusted models, moderate sleep problems (aOR = 2.00, 95% CI: 1.09–3.67, *p* = 0.024) and severe sleep problems (aOR = 3.63, 95% CI: 1.45–9.06, *p* = 0.006) were significantly associated with severe dysmenorrhea. Severe dysmenorrhea strongly predicted menstrual-related functional impairment (aOR = 4.81, 95% CI: 2.63–8.77, *p* < 0.001). Poor sleep quality remained independently associated with functional impairment (aOR = 1.96, 95% CI: 1.05–3.65, *p* = 0.035). In component analysis, sleep disturbance (PSQI Component 5) was the only independent predictor of severe dysmenorrhea (*a*OR = 2.11, 95% CI: 1.31–3.41, *p* = 0.002). **Conclusions:** Poor sleep quality, particularly sleep disturbance, is associated with increased odds of severe dysmenorrhea and menstrual-related functional impairment in female university students. Sleep fragmentation may represent a key mechanistic and modifiable contributor to menstrual pain severity. Integrating sleep assessment into dysmenorrhea management and evaluating sleep-focused interventions in longitudinal and interventional studies are warranted.

## 1. Introduction

Poor sleep quality, whether it is due to short sleeping time, superficial sleep, or interrupted sleep, is prevalent among college students, especially those in academically demanding disciplines such as medicine and health sciences. It is estimated that almost 60% of students in medical and health sciences colleges suffer from poor sleep quality in the Middle East and North Africa regions [1]. Chronic poor sleep can predispose individuals to many health concerns, including impaired cognitive function, increased anxiety and depression, and altered pain perception [2,3,4]. This study aims to assess the prevalence of poor sleep and explore its association with menstrual pain in female students at medical and health sciences colleges.

The link between sleep and pain is supported by biological evidence. It is known that poor sleep can stimulate the release of inflammatory mediators and enhance prostaglandin synthesis, which are directly linked to higher pain perception [5,6]. Additionally, sleep disturbance can disrupt the Hypothalamic–Pituitary–Ovarian (HPO) axis and alter the pulsatility of the gonadotropin-releasing hormone (GnRH), which could potentially affect the reproductive process [7]. Recent cross-sectional studies have reported associations between poor sleep or high stress and menstrual symptoms such as dysmenorrhea, premenstrual syndrome, or irregular cycles in various populations [8,9,10,11]. Some recent studies in the UAE (United Arab Emirates) showed a high prevalence of dysmenorrhea among medical and health sciences students with a significant effect on academic performance [12,13]. Despite this emerging evidence, few studies have explored the effect of chronic poor sleep on the functional impact of dysmenorrhea and resultant impairment (e.g., impact on productivity and daily life). Additionally, high stress is frequently associated with poor sleep and may act as a confounding or mediating factor in the sleep–dysmenorrhea relationship; however, few studies have controlled for this variable.

This study aimed to assess the prevalence of poor sleep quality among female university students and to explore its association with severe dysmenorrhea and menstrual-related functional impairment. The specific objectives of this study were:

Primary Objective: To determine the prevalence of poor sleep quality and to explore its association with the odds of developing severe dysmenorrhea among female university students.

Secondary Objective: To assess whether poor sleep quality is independently associated with increased odds of menstrual-related functional impairment after accounting for dysmenorrhea severity and other confounders.

We formally hypothesized the following associations:

**Primary Hypothesis (Ha,1).** 
*Poor sleep quality is independently associated with increased odds of severe dysmenorrhea among female university students.*

**Secondary Hypothesis (Ha,2).** 
*Poor sleep quality is independently associated with increased odds of significant menstrual-related functional impairment.*

## 2. Materials and Methods

### 2.1. Study Design and Ethical Considerations

This cross-sectional study was conducted among female undergraduate students enrolled at Ras Al Khaimah Medical and Health Sciences University (RAKMHSU), United Arab Emirates. Ethical approval was obtained from the Ras Al Khaimah Research Ethics Committee (RAKREC) (RAKMHSU-HEC-144-UG-M; 27 November 2024). Written informed consent was obtained from all participants prior to data collection.

### 2.2. Participants and Sampling

The target population comprised approximately 700 female students enrolled at Ras Al Khaimah Medical and Health Sciences University (RAKMHSU).

The minimum required sample size (*n*0) was calculated, assuming a 95% confidence level (*Z* = 1.96), a conservative estimated prevalence of 50% (*p* = 0.50), and a desired margin of error of 5% (*d* = 0.05). Using the standard formula for estimating a proportion in an infinite population [14]:

$$n0=\frac{Z^{2}\;.\;\;p\;\left(1-p\right)}{d^{2}}$$

$$n0=\frac{{\left(1.96\right)}^{2}\;.\;\;0.5\;\left(1-0.5\right)}{({0.05)}^{2}}\approx \;385$$

As the target population was finite (*N* ≈ 700), a finite population correction was applied:

$$n=\frac{n0\;}{1+\frac{n0-1}{N}}$$

$$n=\frac{385\;}{1+\frac{385-1}{700}}\approx 249$$

Therefore, the minimum required sample size (*n*) was estimated to be approximately 250 participants.

Convenience sampling was employed during the data collection period (December 2024 to May 2025). All eligible participants who completed the questionnaire were included in the descriptive and prevalence analyses. For analyses examining associations between sleep quality and menstrual outcomes, a restricted analytic sample was defined as described below.

### 2.3. Inclusion Criteria

Participants were eligible for inclusion if they were:

1. Female students actively enrolled at the university.
2. Currently menstruating (e.g., having a menstrual cycle within the last 6 months).
3. Provided written informed consent to participate.

### 2.4. Exclusion Criteria for Association Analyses

Participants were excluded if they reported conditions or treatments independently known to affect sleep quality, pain perception, or menstrual function. The exclusion criteria included any use of hormonal medications such as oral contraceptives, transdermal patches, or intrauterine systems, or other hormonal therapies, either currently or within the preceding three months. Use of medications known to alter sleep–wake regulation, including hypnotics, sedatives, stimulants, antidepressants, antipsychotics, and other centrally acting agents, was also assessed during screening and constituted an exclusion criterion. Students with diagnosed gynecological conditions known to affect menstrual characteristics, such as endometriosis, polycystic ovary syndrome, or uterine fibroids, or diagnosed with major psychiatric disorders, including bipolar disorder or major depressive disorder, or those with fibromyalgia were also excluded. History of pregnancy or breastfeeding within the previous 12 months was also an exclusion criterion. Students with these criteria were included in the descriptive and prevalence analysis but excluded from analyses assessing the association between sleep quality and dysmenorrhea-related outcomes.

### 2.5. Data Collection Tool and Variables

Data collection was accomplished using a self-report questionnaire administered to all participants via an online platform. This instrument comprised standardized, validated scales to measure sleep quality and perceived stress, as well as a series of custom questions designed to capture relevant demographic and health information.

#### 2.5.1. Sleep Quality

The Pittsburgh Sleep Quality Index (PSQI) is a standardized 19-item questionnaire that evaluates sleep quality and related disturbances over the previous month. Rather than focusing on sleep duration alone, it captures multiple dimensions of sleep, including subjective perceptions of sleep quality, difficulty initiating sleep, total sleep duration, habitual sleep efficiency, nocturnal disturbances, use of sleep medication, and impairment in daytime functioning [15]. Each of these domains is represented by a distinct PSQI component (C1–C7), as summarized in Table 1. Each component is scored from 0 (no difficulty) to 3 (severe difficulty), and the sum of the seven components yields a global score ranging from 0 to 21. Higher scores indicate poorer sleep quality. Conventionally, a global score of 0–5 indicates good sleep quality, scores of 6–10 indicate moderate sleep problems, and scores of 11–21 indicate severe sleep problems. Poor sleep quality in this study was defined as a PSQI score > 5 [15,16]. The internal consistency of the PSQI in this study was good (Cronbach’s α = 0.849).

#### 2.5.2. Menstrual Variables

Menstrual outcomes were collected for the most recent menstrual period.

- Dysmenorrhea severity: Dysmenorrhea severity was assessed using a visual analog scale (VAS) ranging from 0 (no pain) to 10 (worst pain imaginable). Scores ≥7 were classified as severe dysmenorrhea, consistent with established clinical thresholds indicating probable functional impairment [17,18].
- Menstrual-related functional impairment: Participants reported the degree of impairment (not at all, slightly, moderately, or severely) in three domains during their most recent menstrual period: social activities, physical activity, and academic or work productivity. Overall functional impairment was analyzed as a dichotomous variable (Yes/No), with “Yes” defined as reporting moderate or severe impairment in at least one domain.
- Other menstrual information: This included cycle length, duration of menstrual bleeding, and menstrual irregularity. Menstrual irregularity was defined as cycles occurring seven or more days earlier or later than expected.

#### 2.5.3. Other Variables

- Demographic and anthropometric data: age, academic program, year of study, self-reported socioeconomic status, and body mass index (BMI), calculated from self-reported height and weight.
- Medical and medication history: Self-reported chronic medical conditions and current or recent medication use.
- Perceived Stress Scale (PSS-10): Perceived stress, a key confounder, was assessed using the 10-item Perceived Stress Scale (PSS-10). This is a validated instrument that evaluates how unpredictable, uncontrollable, and overloaded individuals perceived their lives during the previous month. The scale consists of 10 items: six are negatively worded (e.g., feeling nervous, stressed, or overwhelmed), and four are positively worded (e.g., feeling confident in handling problems) and are reverse-scored. Each item is rated on a 5-point Likert scale ranging from 0 (never) to 4 (very often). The total score ranges from 0 to 40, with higher scores indicating greater perceived stress. Standard cut-offs classify scores of 0–13 as low stress, 14–26 as moderate stress, and 27–40 as high stress. For this study, low stress was defined as a PSS-10 score < 14 [19]. The internal consistency of the PSS-10 was good (Cronbach’s α = 0.832).

### 2.6. Statistical Analysis

Descriptive statistics were used to summarize participant characteristics. Categorical variables, including academic program, socioeconomic status, sleep quality, menstrual cycle regularity, dysmenorrhea severity, and menstrual-related functional impairment, were presented as frequencies and percentages. Continuous variables, including age, BMI, perceived stress score, and sleep quality score, were summarized as median (interquartile range), based on their distribution. Normality of continuous variables was assessed using a combination of the Shapiro–Wilk test, Q–Q plots, skewness and kurtosis values, and visual inspection of histograms.

The prevalence of sleep problems, severe dysmenorrhea, menstrual-related functional impairment, and irregular menstruation was described for the overall sample. The prevalence of menstrual outcomes was also examined according to sleep quality status (good vs. poor sleep). Unadjusted associations between sleep quality and each categorical variable were assessed using Pearson’s chi-square test.

Multivariate logistic regression analyses were performed to estimate adjusted odds ratios (aORs) and 95% confidence intervals (CIs) for the association between poor sleep quality and menstrual variables as follows:

Model 1 evaluated the association between poor sleep quality and severe dysmenorrhea, adjusting for age, BMI, perceived stress level and socioeconomic status.

Model 2 assessed the association between poor sleep quality and menstrual-related functional impairment, adjusting for the same covariates and for the presence of severe dysmenorrhea, as it can be a mediator of functional impairment.

All statistical analyses were conducted using IBM SPSS Statistics for Windows, version 30.0 (IBM Corp., Armonk, NY, USA). A *p*-value of <0.05 was considered statistically significant.

We assessed the study’s sensitivity by calculating the minimum detectable effect (MDE) based on the achieved sample size, outcome prevalence, and α = 0.05. With 80% statistical power, the study was able to detect odds ratios of approximately 2.0 or higher for the associations of interest. Consequently, non-significant findings involving smaller effect sizes should be interpreted with caution, as the study may have been underpowered to detect more modest associations.

## 3. Results

A total of 254 female students completed the questionnaire and were included in the descriptive analyses. Of these, 226 students met the criteria for inclusion in the analytic sample used for association analyses after excluding participants with conditions or treatments known to affect sleep or menstrual function. Exclusions were due to polycystic ovary syndrome (n = 26), current use of combined oral contraceptives (n = 1), and hypothyroidism (n = 1).

In the full descriptive sample (n = 254), the median age was 20.0 years (19–22), the median BMI was 23.1 kg/m^2^ (20.6–26.4), and the median age at menarche was 13.0 years [12,13,15]. The median menstrual cycle length was 28.0 days (28–30), with a median menstrual duration of 6.0 days (5–7). Most participants were enrolled in Medicine programs (MBBS/MD) (53.9%), followed by the Bachelor of Nursing (BSN) program (26.0%), the Bachelor of Pharmacy (B.Pharm) program (11.0%), and the Bachelor of Dental Surgery (BDS) program (9.1%). Participants were distributed across academic years, with approximately half coming from Years 1 and 2. The majority reported a medium socioeconomic status (81.9%), while smaller proportions reported high (16.1%) or low (2.0%) socioeconomic status. Most students (86.1%) reported moderate stress levels, and 10.2% reported high stress. Participant characteristics of the descriptive sample (n = 254) are summarized in Table 2.

### 3.1. Prevalence of Sleep Problems and Menstrual-Related Outcomes

Overall, in the descriptive sample (n = 254), poor sleep quality was reported by 68.9% of participants, with 54.7% experiencing moderate sleep problems and 14.2% experiencing severe sleep problems. Comparisons between participants with poor versus good sleep quality showed no significant differences in age, BMI, menstrual age, cycle length, or PSS score; see Table 3. Comparable findings were observed when these baseline comparisons were repeated in the restricted analytic sample (n = 226). No significant differences were observed between sleep-quality groups in socioeconomic status (χ^2^(2) = 2.32, *p* = 0.313) or year of study (χ^2^(4) = 4.48, *p* = 0.346). In contrast, academic program distribution differed between groups (χ^2^(3) = 8.54, *p* = 0.036), with the highest proportion of poor sleepers observed in the BDS program (87.0%), followed by MBBS/MD (73.0%), BSN (63.6%), and B.Pharm (53.6%), see Supplementary Table S1.

Menstrual-related symptoms were prevalent in the study population. Severe dysmenorrhea was reported by 48.8% of participants, and 30.3% reported irregular menstrual cycles. More than half of the students (58.3%) experienced at least one form of menstrual-related functional impairment. Moderate to severe impairment was most frequently reported in physical activity (48.8%), followed by daily productivity (44.1%) and social activity (43.3%); see Table 2.

### 3.2. Sleep Quality and Menstrual-Related Outcomes

Significant associations were observed between sleep quality and multiple menstrual-related outcomes in the analytic sample (n = 226) (Table 4). Students with poor sleep were significantly more likely to report severe dysmenorrhea, with an odds ratio (OR) of 2.18 (95% CI: 1.22–3.88, *p* = 0.008). Poor sleep was also strongly associated with functional impairment during menstruation, with affected students having nearly threefold higher odds (OR = 2.75, 95% CI: 1.55–4.87, *p* < 0.001). Similarly, poor sleep was significantly associated with reduced physical activity (OR = 2.79, 95% CI: 1.56–4.99, *p* < 0.001) and, to a lesser extent, with reduced social activity (OR = 1.83, 95% CI: 1.02–3.27, *p* = 0.041). Students with poor sleep also had higher odds of reduced daily productivity (OR = 2.36, 95% CI: 1.31–4.27, *p* = 0.004). In contrast, irregular menses did not differ significantly by sleep quality (OR = 1.06, 95% CI: 0.58–1.96).

A multivariate logistic regression was conducted to examine the association between sleep quality and severe dysmenorrhea after adjusting for age, BMI, socioeconomic status, and PSS-10 score. Participants with poor sleep (PSQI > 5) had 2.5 times higher odds of severe dysmenorrhea compared with those reporting good sleep quality (PSQI ≤ 5) (aOR = 2.49, 95% CI: 1.37–4.50, *p* = 0.003); see Table 5a. A secondary model examined levels of sleep problems to explore dose–response effects. Compared with good sleep, moderate sleep problems (PSQI 6–10) were associated with a twofold increase in the odds of severe dysmenorrhea (aOR = 2.00, 95% CI: 1.09–3.67, *p* = 0.024), and severe sleep problems (PSQI ≥ 11) with a more than threefold increase (aOR = 3.63, 95% CI: 1.45–9.06, *p* = 0.006).

Higher perceived stress showed a trend toward an association with severe dysmenorrhea; each one-point increase in PSS-10 score was associated with a 6% increase in the odds of severe dysmenorrhea (aOR = 1.06, 95% CI: 1.00–1.13, *p* = 0.053). Age, BMI, and socioeconomic status were not significant predictors.

A second multivariate logistic regression was conducted to assess the association between poor sleep and menstrual-related functional impairment while controlling for severe dysmenorrhea, stress level, and demographic variables. Severe dysmenorrhea was the strongest predictor, with affected participants having more than fourfold higher odds of functional impairment (aOR = 4.81, 95% CI: 2.63–8.77, *p* < 0.001) compared to unaffected participants. Poor sleep remained significantly associated (aOR = 1.96, 95% CI: 1.05–3.65, *p* = 0.035); see Table 5b. In contrast, age, BMI, economic status, and stress were not significant. Although the effect of poor sleep was significant, the result is borderline due to the CI’s lower bound being very close to the null value of 1.0. This suggests that the precision of this estimate is low, and the size of the independent effect might be quite small.

A multivariate logistic regression model was conducted to examine whether PSQI components were independently associated with severe dysmenorrhea after adjustment for age, BMI, socioeconomic status, and stress level. Sleep disturbances (Component 5) emerged as the only significant independent predictor. Each one-point increase in disturbance score was associated with a twofold increase in the odds of severe dysmenorrhea (aOR = 2.11, 95% CI: 1.31–3.41, *p* = 0.002). Although subjective sleep quality (Component 1) and sleep latency (Component 2) showed significant associations in unadjusted analyses, these relationships did not persist once the components were entered together. This pattern indicates that sleep fragmentation is the dimension most strongly associated with dysmenorrhea severity, see Supplementary Table S2.

## 4. Discussion

This study examined the relationship between sleep quality and menstrual health outcomes among female university students. Three key findings emerged. First, poor sleep quality was highly prevalent and was associated with significantly increased odds of severe dysmenorrhea in both unadjusted and adjusted models. Second, when individual sleep dimensions were analyzed concurrently, sleep disturbance—rather than subjective sleep quality, sleep latency, or daytime dysfunction—was the only PSQI component that was independently associated with severe dysmenorrhea. This finding suggests that fragmented or disrupted sleep may represent the strongest sleep dimension associated with menstrual pain severity. Third, both poor sleep quality and severe dysmenorrhea were independently associated with greater menstrual-related functional impairment. However, after adjustment for severe dysmenorrhea, poor sleep quality demonstrated a comparatively smaller effect size, suggesting that menstrual pain may play a more dominant role in functional limitation.

The prevalence of poor sleep quality (68.9%) and severe dysmenorrhea (48.8%) observed in this cohort is consistent with regional and international literature documenting a high burden of sleep problems and menstrual morbidity among medical and health sciences students [1,12,13]. A recent study from the United Arab Emirates similarly reported a high prevalence of menstrual symptoms and associated functional impairment, highlighting the regional burden of menstrual morbidity [20]. Comparable studies from Saudi Arabia and Ethiopia have also identified dysmenorrhea as a contributor to absenteeism and reduced academic performance among female students [21,22]. In addition, a systematic review by Maity et al. demonstrated that menstrual disturbances adversely affect quality of life and academic outcomes among medical students [23]. The present study extends this evidence base by identifying sleep quality as a potentially modifiable factor associated with dysmenorrhea severity and related impairment.

The observed association between poor sleep quality and dysmenorrhea is consistent with prior epidemiological evidence across adolescent and university populations. In Korean high-school girls assessed with the PSQI, Jeong et al. found that sleep timing variables (late bedtime/wake time) showed crude associations with menstrual symptom severity, but these associations were no longer significant after adjustment for covariates; in contrast, overall sleep quality and several PSQI components remained significantly associated with dysmenorrhea symptom frequency and severity, and multivariable models identified sleep quality as the most influential sleep-related factor among timing, duration, and quality metrics [8]. Complementing these findings, a large population-based study from Upper Egypt distinguished between sleep duration and insomnia symptoms: shorter sleep duration was not associated with dysmenorrhea, whereas insomnia was associated with higher odds of dysmenorrhea (OR 2.6, 95% CI: 1.6–4.3) and with a broad range of premenstrual symptoms [24]. This pattern is compatible with the interpretation that dysmenorrhea may be more strongly linked to insomnia-like features (e.g., difficulty initiating/maintaining sleep and sleep fragmentation) than to sleep duration per se, and it supports examining sleep quality dimensions rather than relying solely on hours slept.

Beyond pain severity, the functional implications observed in the current study align with university-based data showing that menstrual disorders, particularly dysmenorrhea, are associated with clinically and practically meaningful decrements in well-being and academic participation. Among female undergraduates at Makerere University, dysmenorrhea was highly prevalent and was associated with significantly poorer quality-of-life scores, and class absenteeism was reported exclusively among participants with dysmenorrhea [25]. Collectively, these studies support the view that disturbed sleep is linked not only to dysmenorrhea but also to downstream functional consequences, reinforcing the importance of considering sleep quality in strategies aimed at mitigating menstrual morbidity in student populations. From a practical clinical perspective, the magnitude of the observed associations suggests that sleep disturbance and dysmenorrhea frequently coexist at clinically meaningful levels, affecting daily functioning. In student populations, this may translate into a higher likelihood of activity restriction, reduced concentration, and impaired academic engagement during symptomatic periods. Although we cannot determine cause and effect, the findings suggest that sleep disturbance and menstrual pain commonly occur together and may jointly contribute to symptom burden and functional limitation in this population. This supports interpreting sleep disturbance as part of the overall symptom profile reported by some students with dysmenorrhea.

Several biological mechanisms may plausibly underlie the association between sleep disturbance and dysmenorrhea. Dysmenorrhea is characterized by elevated prostaglandin production, particularly prostaglandin F2α and prostaglandin E2, which increase uterine contractility, ischemia, and pain [26]. Sleep disruption has been shown to heighten systemic inflammation, with increases in pro-inflammatory markers such as interleukin-6, tumor necrosis factor-α, and C-reactive protein, potentially lowering pain thresholds and amplifying nociceptive sensitivity [27]. In parallel, chronic sleep disruption adversely affects central pain modulation by impairing descending inhibitory pathways, thereby enhancing pain perception and reducing pain tolerance [28]. These inflammatory and neurophysiological mechanisms provide biologically plausible explanations for the observed association between poor sleep quality and more severe menstrual pain.

Endocrine mechanisms may further contribute to this relationship. Sleep disturbance and psychosocial stress can influence hypothalamic–pituitary–ovarian axis regulation and gonadotropin pulsatility, potentially altering uterine contractility and pain modulation [9]. Sleep and circadian rhythms interact bidirectionally with sex hormone regulation, particularly estrogen and progesterone [29], and fluctuations in sex steroid levels have been associated with variations in PSQI-measured sleep quality [30]. Although the present study cannot establish temporal directionality, these neuroendocrine pathways offer additional explanatory context for the observed associations.

The component-level analysis provides further mechanistic insight. Among the seven PSQI dimensions, sleep disturbance (C5) emerged as the strongest independent predictor of severe dysmenorrhea. This finding is consistent with evidence indicating that sleep fragmentation has been associated with alterations in inflammatory homeostasis and nociceptive processing [27]. However, given the cross-sectional design, the direction of this relationship should be interpreted cautiously. It is equally plausible that severe menstrual pain contributes to nocturnal awakenings and fragmented sleep, thereby increasing sleep disturbance scores. From this perspective, the observed association may reflect, at least in part, the impact of pain on sleep continuity rather than sleep disturbance acting solely as an upstream causal factor.

Higher perceived stress showed a trend toward an association with greater odds of severe dysmenorrhea in adjusted models. Prior studies among university students and healthcare trainees have similarly reported associations between psychological stress, sleep disturbance, and menstrual symptoms. Prior studies among university students and healthcare trainees have similarly reported associations between psychological stress, sleep disturbance, and menstrual symptoms [10,31,32]. Notably, the persistence of sleep quality as an independent predictor in adjusted models suggests that sleep-related pathways may contribute to dysmenorrhea severity at least partly independently of perceived stress.

This study has several strengths, including the use of validated instruments (PSQI, PSS-10, and VAS), adjustment for multiple confounders, and detailed analysis of individual sleep components. The inclusion of menstrual-related functional impairment as an outcome further enhances the clinical and public health relevance of the findings.

Several limitations should be acknowledged. The use of convenience sampling may limit generalizability, and reliance on self-reported measures introduces the potential for recall or reporting bias. Future studies should incorporate objective sleep measures, such as actigraphy or polysomnography, to improve measurement precision and reduce reporting bias. In addition, PSQI Component 5 includes a subitem assessing sleep disruption due to pain. This may introduce partial measurement overlap between sleep disturbance and dysmenorrhea severity, and, therefore, the component-level findings should be interpreted cautiously. The cross-sectional design precludes causal inference, and residual confounding by unmeasured lifestyle or psychosocial factors, such as physical activity and dietary patterns, and use of central nervous system stimulants (e.g., caffeine and nicotine), cannot be excluded. In addition, although overall perceived stress was measured using a validated scale, acute situational stressors coinciding with a specific menstrual cycle (e.g., examination periods or academic deadlines) were not specifically assessed and may have resulted in residual confounding. Although the sample size achieved was sufficient to detect moderate associations, smaller effects may have been underpowered. Consequently, non-significant findings, including the absence of an association between sleep quality and menstrual irregularity, should be interpreted cautiously. Objective sleep measures such as actigraphy or polysomnography were not employed; however, the PSQI remains a widely used and validated tool in sleep and reproductive health research [15,16,33].

In conclusion, the present findings highlight sleep quality, particularly sleep disturbance, as a potentially modifiable factor relevant to menstrual health among university students. University health services may benefit from incorporating sleep screening into routine reproductive health assessments. Interventions aimed at improving sleep hygiene, reducing nocturnal sleep fragmentation, managing stress, and stabilizing circadian rhythms may offer meaningful benefits at relatively low cost. Longitudinal and interventional studies are warranted to determine whether interventions targeting sleep quality influence dysmenorrhea severity or whether effective pain control may improve sleep continuity.

## 5. Conclusions

Chronic poor sleep quality is associated with an increased risk of severe dysmenorrhea and greater menstrual-related functional impairment among female university students. Sleep disturbance, rather than sleep duration alone, appears to be the sleep dimension most strongly associated with symptom severity. These findings suggest that incorporating sleep health assessment into the clinical evaluation of dysmenorrhea warrants consideration. Prospective and interventional studies are needed to establish temporal directionality and determine whether targeted sleep optimization can reduce dysmenorrhea severity and related functional impairment. Interventional trials incorporating cognitive behavioral therapy for insomnia with menstrual symptom outcomes would be particularly informative. Future research should also integrate polysomnography- or actigraphy-based assessments to strengthen objective phenotyping of sleep disturbance.

## Figures and Tables

**Table 1:** Components of the Pittsburgh Sleep Quality Index (PSQI).

Component	Domain	Description
C1	Subjective sleep quality	Individual’s overall perception of sleep quality
C2	Sleep latency	Time taken to fall asleep
C3	Sleep duration	Total number of hours slept per night
C4	Habitual sleep efficiency	Ratio of total sleep time to time spent in bed
C5	Sleep disturbances	Frequency of nocturnal interruptions due to discomfort, illness, or environmental factors
C6	Use of sleep medication	Frequency of sleeping pill use
C7	Daytime dysfunction	Degree of impairment in daily activities due to poor sleep

**Table 2:** Characteristics of the study population (n = 254).

Characteristic	Category/Statistic	Value *
Age (years)	–	20.0 (19.0–22.0)
Body mass index (kg/m^2^)	–	23.1 (20.6–26.4)
Age at menarche (years)	–	13.0 (12.0–14.0)
Menstrual cycle length (days)	–	28.0 (28.0–30.0)
Duration of menstruation (days)	–	6.0 (5.0–7.0)
Menstrual pain score (VAS)	–	6.0 (5.0–8.0)
Perceived stress (PSS-10)	–	20.0 (18.0–23.0)
Sleep quality (PSQI)	–	7.0 (5.0–9.0)
Academic program	MBBS/MD	137 (53.9)
	BSN	66 (26.0)
	B.Pharm	28 (11.0)
	BDS	23 (9.1)
Academic year	Year 1	58 (22.8)
	Year 2	69 (27.2)
	Year 3	50 (19.7)
	Year 4	28 (11.0)
	Year 5	49 (19.3)
Economic status	Low	5 (2.0)
	Moderate	208 (81.9)
	High	41 (16.1)
Severe dysmenorrhea	No	130 (51.2)
	Yes	124 (48.8)
Menstrual-related functional impairment	No	106 (41.7)
	Yes	148 (58.3)
Menstrual-related physical impairment (e.g., physical chores, or exercise)	Not at all	32 (12.6)
	Slight	98 (38.6)
	Moderate	78 (30.7)
	Severe	46 (18.1)
Menstrual-related social impairment (e.g., missing social gatherings, or reduced time with family and friends during menstrual period)	Not at all	49 (19.3)
	Slight	95 (37.4)
	Moderate	76 (29.9)
	Severe	34 (13.4)
Menstrual-related reduced productivity (e.g., physical chores, or exercise)	Not at all	41 (16.1)
	Slight	101 (39.8)
	Moderate	78 (30.7)
	Severe	34 (13.4)
Irregular menstruation	No	177 (69.7)
	Yes	77 (30.3)
Sleep quality category	Good quality	79 (31.1)
	Moderate problem	139 (54.7)
	Severe problem	36 (14.2)
Stress level category	Low	9 (3.5)
	Moderate	221 (87.0)
	High	24 (9.4)

All values in the table refer to the full descriptive sample (n = 254). * Continuous variables are presented as median (interquartile range) while categorical variables are presented as n (%). BMI = body mass index; VAS = visual analog scale; PSS-10 = Perceived Stress Scale-10; PSQI = Pittsburgh Sleep Quality Index; MBBS = Bachelor of Medicine, Bachelor of Surgery; MD = Doctor of Medicine; BDS = Bachelor of Dental Surgery; BPharm = Bachelor of Pharmacy; BSN = Bachelor of Science in Nursing.

**Table 3:** Comparison of continuous variables between good and poor sleep groups in the full descriptive sample (n = 254).

Variable	Good Sleep (n = 77) Median [IQR]	Poor Sleep (n = 177) Median [IQR]	Mann–Whitney U	*p*
Age (years)	20.0 [19.0–21.0]	20.0 [19.0–22.0]	6458	0.501
BMI (kg/m^2^)	22.0 [20.6–25.2]	23.7 [20.6–26.7]	6022	0.141
Menarche age (years)	13.0 [12.0–13.0]	12.5 [12.0–14.0]	6883	0.670
Cycle length (days)	28.0 [28.0–30.0]	28.0 [28.0–30.0]	4899	0.742
Menstrual length (days)	6.0 [5.0–7.0]	6.0 [5.0–7.0]	7020	0.696
PSS-10 score	20.0 [18.0–22.0]	20.0 [19.0–23.0]	6125	0.196

Note. Mann–Whitney U test used due to non-normality. IQR = interquartile range; BMI = Body mass index; PSS = Perceived Stress Scale-10.

**Table 4:** Association between sleep quality and menstrual variables in the analytic sample (n = 226).

Outcome Variable	Good Sleep (n = 74) No/Yes	Poor Sleep (n = 152) No/Yes	χ^2^(1)	*p*	Cramer’s V	Odds Ratio (95% CI)
Severe Dysmenorrhea	49/25	72/80	7.11	0.008	0.177	2.18 (1.22, 3.88)
Overall menstrual-related impairment	43/31	51/101	12.35	<0.001	0.234	2.75 (1.55, 4.87)
Menstrual-related physical impairment	50/24	65/87	12.25	<0.001	0.233	2.79 (1.56, 4.99)
Menstrual-related social impairment	50/24	81/71	4.16	0.041	0.136	1.83 (1.02, 3.27)
Menstrual-related reduced productivity	52/22	76/76	8.33	0.004	0.192	2.36 (1.31, 4.27)
Irregular menstruation	53/21	107/45	0.04	0.849	0.013	1.06 (0.58, 1.96)

χ^2^(1) = Chi-Square statistic; CI = Confidence Interval Odds Ratios are calculated for the Poor Sleep group relative to the Good Sleep group. Analyses in this table are restricted to participants meeting criteria for the analytic sample after exclusion of conditions and treatments affecting sleep or menstrual function.

**Table 5:** Multivariate logistic regression analysis.

**Predictor**	**B**	**SE**	**aOR**	**95% CI**	***p***
a. Significant predictors of severe dysmenorrhea					
**PSS-10 score**	0.062	0.032	1.06	0.99–1.13	0.053
**Poor Sleep** (Yes vs. No)	0.912	0.303	2.49	1.37–4.50	0.003
b. Significant predictors of menstrual-related functional impairment					
**Predictor**	**B**	**SE**	**aOR**	**95% CI**	***p***
**Severe Dysmenorrhea** (Yes vs. No)	1.570	0.307	4.81	2.63–8.77	<0.001
**Poor Sleep** (Yes vs. No)	0.672	0.318	1.96	1.05–3.65	0.035

Model adjusted for age, BMI, socioeconomic status, and PSS-10 score. B = unstandardized regression coefficient; SE = standard error; aOR = adjusted odds ratio; CI = confidence interval. Model adjusted for age. Analyses are restricted to participants meeting criteria for the analytic sample after exclusion of conditions and treatments affecting sleep or menstrual function (n = 226).

## Data Availability

The data presented in this study are available on request from the corresponding author due to participant confidentiality and ethical restrictions related to human subject data.

## Supplementary Materials

The following supporting information can be downloaded at https://www.mdpi.com/article/10.3390/healthcare14040474/s1. Table S1: Comparison of Academic and Socioeconomic Characteristics Between Good and Poor Sleep Quality Groups; Table S2: Multivariable Logistic Regression Model Including All PSQI Sleep Components Predicting Severe Dysmenorrhea.

## Abbreviations

The following abbreviations are used in this manuscript:

aOR	Adjusted Odds Ratio
BMI	Body Mass Index
BDS	Bachelor of Dental Surgery
B.Pharm	Bachelor of Pharmacy
BSN	Bachelor of Science in Nursing
CI	Confidence Interval
GnRH	Gonadotropin-Releasing Hormone
HPO Axis	Hypothalamic–Pituitary–Ovarian Axis
IL-6	Interleukin-6
MD	Doctor of Medicine
MDE	Minimum Detectable Effect
OR	Odds Ratio
PCOS	Polycystic Ovary Syndrome
PSS-10	Perceived Stress Scale–10
PSQI	Pittsburgh Sleep Quality Index
RAKMHSU	Ras Al Khaimah Medical and Health Sciences University
SPSS	Statistical Package for the Social Sciences
TNF-α	Tumor Necrosis Factor-Alpha
UAE	United Arab Emirates
VAS	Visual Analog Scale
