# Supplementary material for: Prevalence of Poor Sleep Quality and Its Association with Dysmenorrhea Among Female Undergraduate Students at a Health Sciences University in the UAE
Source: Healthcare (Basel). 2026 Feb 13;14(4):474. doi: 10.3390/healthcare14040474 (PMC12941023; doi:10.3390/healthcare14040474)
Supplement: Supplementary file 1 [file healthcare-14-00474-s001.zip › healthcare-4094262-supplementary.pdf]

**Supplementary Materials:**

**Supplementary Table S1.** Comparison of Academic and Socioeconomic Characteristics Between Good and Poor Sleep Quality Groups

| Characteristic | Category | Good Sleep (n = 77) | Poor Sleep (n = 177) | Total (N = 254) | p |
|---|---|---|---|---|---|
| Academic year | Year 1 | 19 (24.7) | 39 (22.0) | 58 (22.8) | |
| | Year 2 | 24 (31.2) | 45 (25.4) | 69 (27.2) | |
| | Year 3 | 14 (18.2) | 36 (20.3) | 50 (19.7) | |
| | Year 4 | 4 (5.2) | 24 (13.6) | 28 (11.0) | |
| | Year 5 | 16 (20.8) | 33 (18.6) | 49 (19.3) | .346† |
| Socioeconomic status | Low | 3 (3.9) | 2 (1.1) | 5 (2.0) | |
| | Moderate | 63 (81.8) | 145 (81.9) | 208 (81.9) | |
| | High | 11 (14.3) | 30 (16.9) | 41 (16.1) | .313† |
| Academic program | B.Pharm | 13 (16.9) | 15 (8.5) | 28 (11.0) | |
| | BDS | 3 (3.9) | 20 (11.3) | 23 (9.1) | |
| | BSN | 24 (31.2) | 42 (23.7) | 66 (26.0) | |
| | MBBS / MD | 37 (48.1) | 100 (56.5) | 137 (53.9) | **.036†** |

Note. Data are presented as n (%). Sleep quality groups were defined using the Pittsburgh Sleep Quality Index (PSQI), with scores ≤5 classified as good sleep and >5 as poor sleep. Group differences were assessed using Chi-square tests.
†Chi-square test.

MBBS = Bachelor of Medicine, Bachelor of Surgery; MD = Doctor of Medicine; BDS = Bachelor of Dental Surgery; BPharm = Bachelor of Pharmacy; BSN = Bachelor of Science in Nursing

**Supplementary Table S2.** Multivariable Logistic Regression Model Including All PSQI Sleep Components Predicting Severe Dysmenorrhea

| Predictor | B | SE | OR 95% CI | | p |
|---|---|---|---|---|---|
| Subjective sleep quality (C1) | 0.214 | 0.214 | 1.238 | 0.81–1.89 | .319 |
| Sleep latency (C2) | 0.265 | 0.179 | 1.304 | 0.92–1.85 | .138 |
| Sleep duration (C3) | 0.007 | 0.164 | 1.007 | 0.73–1.39 | .965 |
| Habitual sleep efficiency (C4) | 0.134 | 0.224 | 1.143 | 0.74–1.77 | .550 |
| Sleep disturbances (C5) | 0.748 | 0.244 | 2.113 | 1.31-3.41 | .002 |
| Use of sleep medication (C6) | -0.067 | 0.207 | 0.935 | 0.62–1.40 | .747 |
| Daytime dysfunction (C7) | -0.050 | 0.191 | 0.952 | 0.65-1.38 | .796 |

Note. Results are from a single multivariable binary logistic regression model in which all PSQI sleep components (subjective sleep quality, sleep latency, sleep disturbances, and daytime dysfunction) were entered simultaneously to predict severe dysmenorrhea. The model was adjusted for age, body mass index (BMI), socioeconomic status, and perceived stress score-10 (PSS-10). B = unstandardized regression coefficient; SE = standard error; OR = odds ratio; CI = confidence interval. p values < .05 were considered statistically significant.
